# A guide to selecting high-performing antibodies for STXBP1 (UniProt ID: P61764) for use in western blot, immunoprecipitation, and immunofluorescence

**DOI:** 10.12688/f1000research.160174.1

**Published:** 2024-12-24

**Authors:** Riham Ayoubi, Charles Alende, Maryam Fotouhi, Sara González Bolívar, Carl Laflamme, NeuroSGC/YCharOS/EDDU collaborative group, ABIF consortium

**Affiliations:** 1 Neurology and Neurosurgery, Montreal Neurological Institute-Hospital, Montreal, Québec, Canada

**Keywords:** P61764, STXBP1, Syntaxin-binding protein 1, MUNC18-1, antibody characterization, antibody validation, western blot, immunoprecipitation, immunofluorescence

## Abstract

The Syntaxin-binding protein 1, STXBP1, is a protein involved in docking and fusion of synaptic vesicles, a crucial event for neurotransmitters release into the synapse. Here we have characterized twelve STXBP1 commercial antibodies for western blot, immunoprecipitation, and immunofluorescence using a standardized experimental protocol based on comparing read-outs in knockout cell lines and isogenic parental controls. These studies are part of a larger, collaborative initiative seeking to address antibody reproducibility issues by characterizing commercially available antibodies for human proteins and publishing the results openly as a resource for the scientific community. While use of antibodies and protocols vary between laboratories, we encourage readers to use this report as a guide to select the most appropriate antibodies for their specific needs.

## Introduction

SNARE proteins are synaptic-soluble N-ethylmaleimide-sensitive factor attachment receptors. Upon its assembly with Syntaxin 1 and other SNARE proteins, STXBP1 forms a tight complex that drives the fusion of synaptic vesicles with the presynaptic plasma membrane, allowing the exocytosis of neurotransmitters. ^1^ STXBP1 haploinsufficiency is one of the most common genetic causes of developmental and epileptic encephalopathies. ^2^ Identification of efficient research tools such as antibodies enables fundamental cell-based assays to understand neurological disorders related to this protein.

This research is part of a broader collaborative initiative in which academics, funders and commercial antibody manufacturers are working together to address antibody reproducibility issues by characterizing commercial antibodies for human proteins using standardized protocols, and openly sharing the data. ^3– 5^ Here we evaluated the performance of twelve commercial antibodies for STXBP1 for use in western blot, immunoprecipitation, and immunofluorescence, enabling biochemical and cellular assessment of STXBP1 properties and function. The platform for antibody characterization used to carry out this study was endorsed by a committee of industry academic representatives. It consists of identifying human cell lines with adequate target protein expression and the development/contribution of equivalent knockout (KO) cell lines, followed by antibody characterization procedures using most commercially available antibodies against the corresponding protein. The standardized consensus antibody characterization protocols are openly available on Protocol Exchange, a preprint server (DOI: 10.21203/rs.3.pex-2607/v1). ^6^

The authors do not engage in result analysis or offer explicit antibody recommendations. Our primary aim is to deliver top-tier data to the scientific community, grounded in Open Science principles. This empowers experts to interpret the characterization data independently, enabling them to make informed choices regarding the most suitable antibodies for their specific experimental needs. Guidelines on how to interpret antibody characterization data found in this study are featured on the YCharOS gateway. ^7^

## Results and discussion

Our standard protocol involves comparing readouts from WT (wild type) and KO cells. ^8, 9^ The first step was to identify a cell line(s) that expresses sufficient levels of a given protein to generate a measurable signal using antibodies. To this end, we examined the DepMap (Cancer Dependency Map Portal, RRID:SCR_017655) transcriptomics database to identify all cell lines that express the target at levels greater than 2.5 log _2_ (transcripts per million “TPM” + 1), which we have found to be a suitable cut-off. ^3^ The U-87 MG expresses the *STXBP1* transcript at 5.2, which is above the average range of cancer cells analyzed. *STXBP1* KO cells in U-87 MG were custom made at Abcam ( Table 1).

**Table 1.** Summary of the cell lines used.

Institution	Catalog number	RRID (Cellosaurus)	Cell line	Genotype
ATCC	HTB-14	CVCL_0022	U-87 MG	WT
Abcam	ab322392	CVCL_E4AP	U-87 MG	*STXBP1* KO

**Figure 1.**
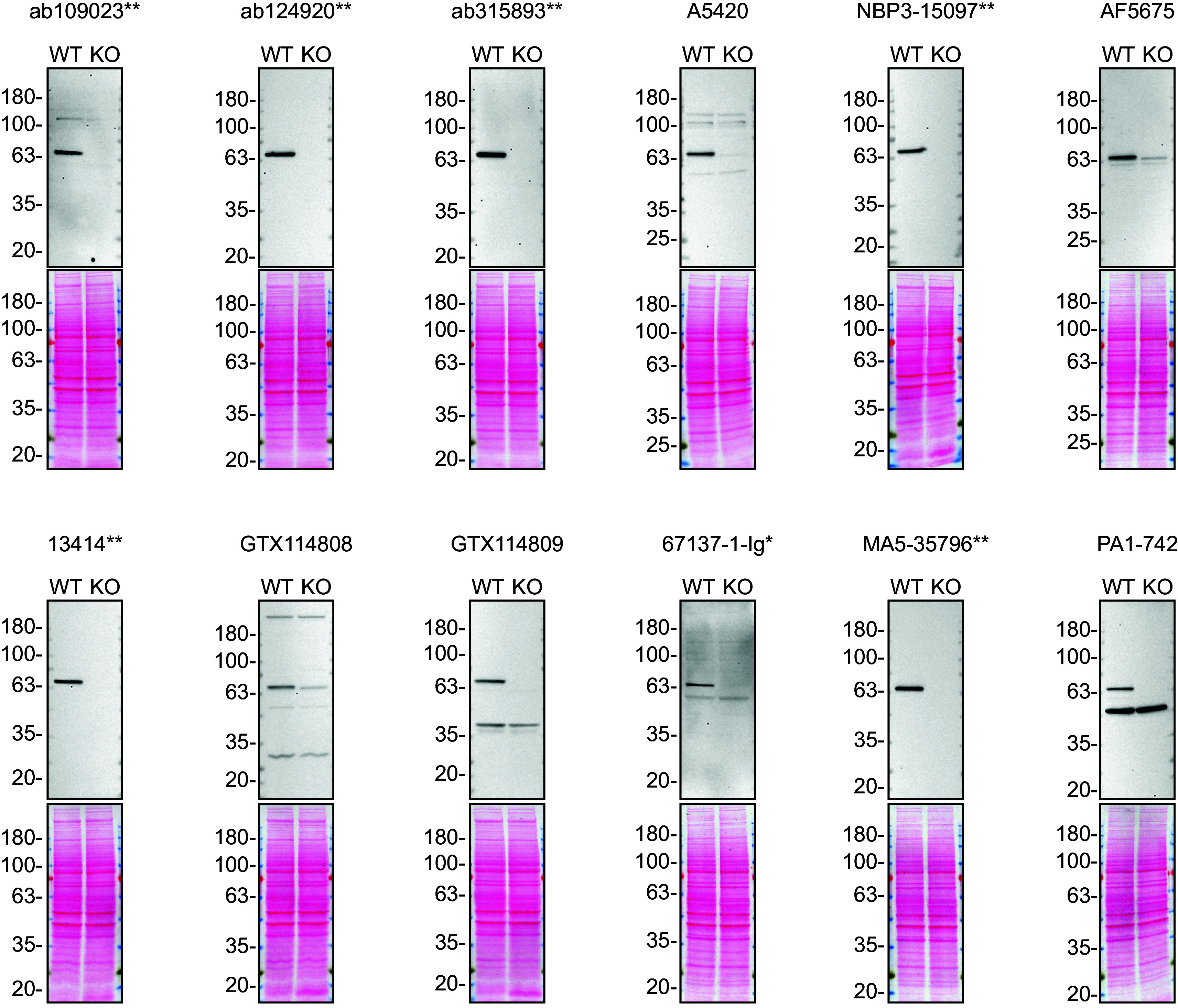
STXBP1 antibody screening by western blot. Lysates of U-87 MG WT and *STXBP1* KO were prepared, and 40 μg of protein were processed for western blot with the indicated STXBP1 antibodies. The Ponceau stained transfers of each blot are presented to show equal loading of WT and KO lysates and protein transfer efficiency from the acrylamide gels to the nitrocellulose membrane. Antibody dilutions were chosen according to the recommendations of the antibody supplier. Antibody dilution used: ab109023** at 1/1000, ab124920** at 1/10 000, ab315893** at 1/1000, A5420 at 1/1000, NBP3-15097** at 1/2000, AF5675 at 1/200, 13414** at 1/1000, GTX114808 at 1/500, GTX114809 at 1/500, 67137-1-Ig* at 1/5000, MA5-35796** at 1/2000, PA1-742 at 1/500 (2 μg/ml). Predicted band size: 67.5 kDa. **Recombinant antibody, *Monoclonal antibody.

To screen all twelve antibodies by western blot, WT and *STXBP1* KO protein lysates were ran on SDS-PAGE, transferred onto nitrocellulose membranes, and then probed with all STXBP1 antibodies in parallel ( Figure 1).

**Figure 2.**
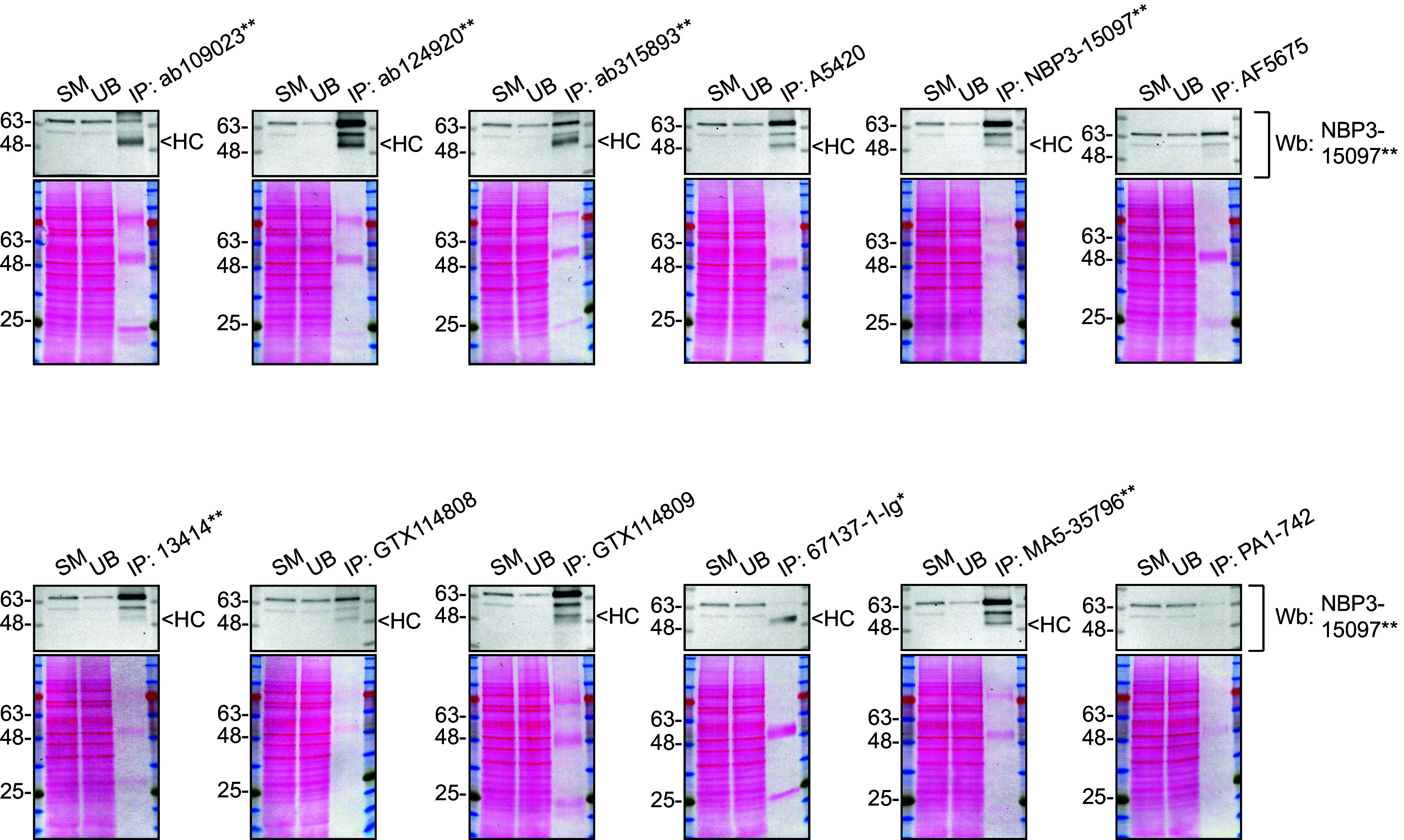
STXBP1 antibody screening by immunoprecipitation. U-87 MG lysates were prepared, and immunoprecipitation was performed using 0.6 mg of lysate and 2.0 μg of the indicated STXBP1 antibodies pre-coupled to Dynabeads protein A or protein G. Samples were washed and processed for western blot with the indicated STXBP1 antibody. For western blot, NBP3-15097** was used at 1/2000. The Ponceau stained transfers of each blot are shown. SM=4% starting material; UB=4% unbound fraction; IP=immunoprecipitate, HC= antibody heavy chain. **Recombinant antibody, *Monoclonal antibody.

We then assessed the capability of the twelve antibodies to capture STXBP1 from U-87 MG protein extracts using immunoprecipitation followed by western blot analysis. For the immunoblot step, a specific STXBP1 antibody identified previously (refer to Figure 1) was selected. Equal amounts of the starting material (SM) and the unbound fractions (UB), as well as the whole immunoprecipitate (IP) eluates were separated by SDS-PAGE ( Figure 2).

**Figure 3.**
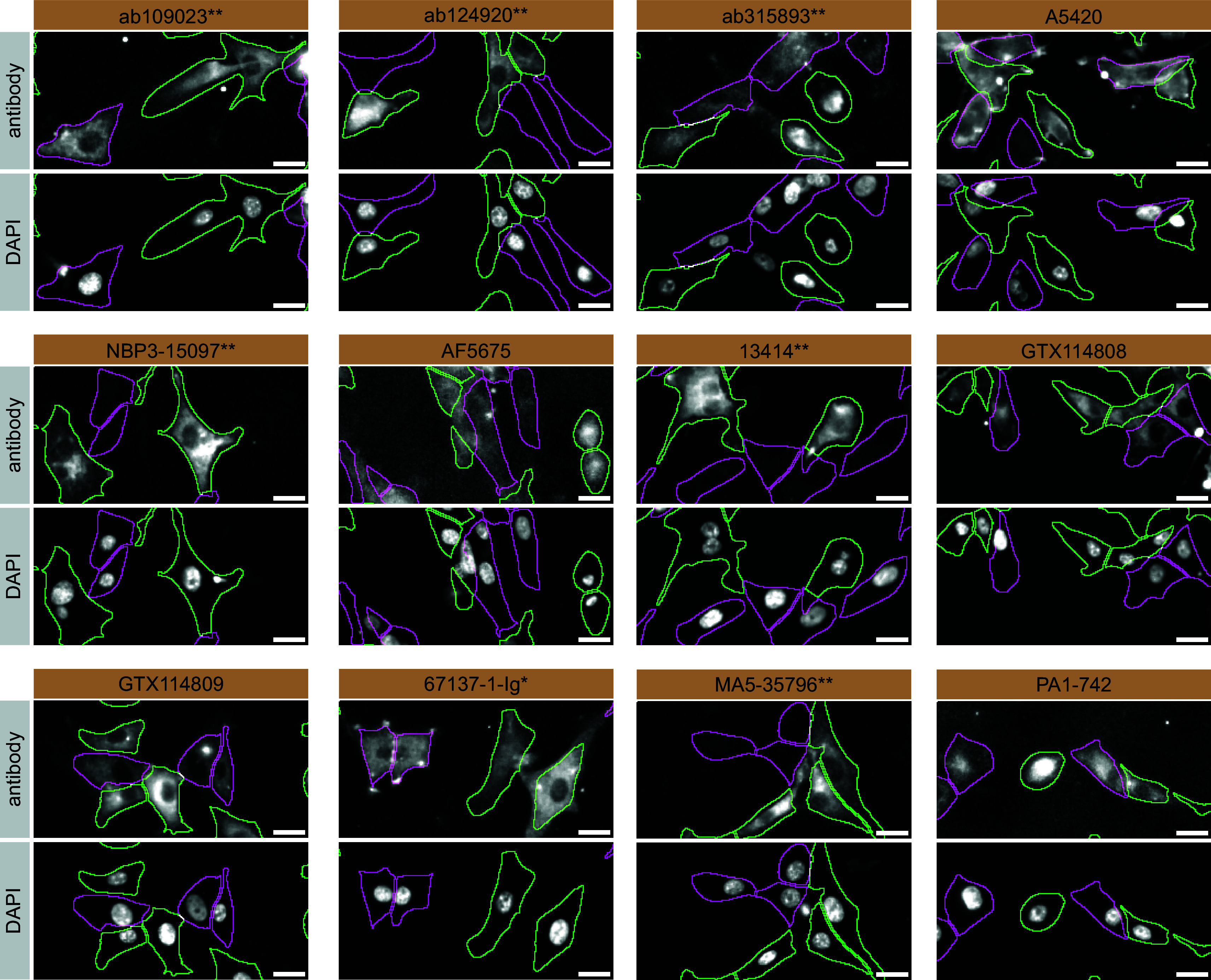
STXBP1 antibody screening by immunofluorescence. U-87 MG WT and *STXBP1* KO cells were labelled with a green or a far-red fluorescent dye, respectively. WT and KO cells were mixed and plated to a 1:1 ratio on coverslips. Cells were stained with the indicatedSTXBP1 antibodies and with the corresponding Alexa-fluor 555 coupled secondary antibody including DAPI. Acquisition of the blue (nucleus-DAPI), green (WT), red (antibody staining) and far-red (KO) channels was performed. Representative images of the merged blue and red (grayscale) channels are shown. WT and KO cells are outlined with green and magenta dashed line, respectively. When an antibody was recommended for immunofluorescence by the supplier, we tested it at the recommended dilution. The rest of the antibodies were tested at 1 and 2 μg/ml and the final concentration was selected based on the detection range of the microscope used and a quantitative analysis not shown here. Antibody dilution used: ab109023** at 1/100, ab124920** at 1/100, ab315893** at 1/500, A5420 at 1/50, AF5675 at 1/200, NBP3-15097** at 1/500, 13414** at 1/100, GTX114808 at 1/800, GTX114809 at 1/1000, 67137-1-Ig* at 1/2000, MA5-35796** at 1/100, PA1-742 at 1/1000. Bars = 10 μm. **Recombinant antibody, *Monoclonal antibody.

For immunofluorescence, the twelve antibodies were screened using a mosaic strategy. First, U-87 MG WT and *STXBP1* KO cells were labelled with different fluorescent dyes in order to distinguish the two cell lines, and the STXBP1 antibodies were evaluated. Both WT and KO lines imaged in the same field of view to reduce staining, imaging and image analysis bias ( Figure 3). Quantification of immunofluorescence intensity in hundreds of WT and KO cells was performed for each antibody tested, and the images presented in Figure 3 are representative of this analysis. ^6^

In conclusion, we have screened twelve STXBP1 commercial antibodies by western blot, immunoprecipitation, and immunofluorescence by comparing the signal produced by the antibodies in human U-87 MG WT and *STXBP1* KO cells. To assist users in interpreting antibody performanyce, Table 3 outlines various scenarios in which antibodies may perform in all three applications. ^3^ Several high-quality and renewable antibodies that successfully detect STXBP1 were identified in all applications. Researchers who wish to study STXBP1 in a different species are encouraged to select high-quality antibodies, based on the results of this study, and investigate the predicted species reactivity of the manufacturer before extending their research.

The underlying data for this study can be found on Zenodo, an open-access repository for which YCharOS has its own collection of antibody characterization reports. A link is accessible in the “Data availability” portion of this data note.

### Limitations

Inherent limitations are associated with the antibody characterization platform used in this study. Firstly, the YCharOS project focuses on renewable (recombinant and monoclonal) antibodies and does not test all commercially available STXBP1 antibodies. YCharOS partners provide approximately 80% of all renewable antibodies, but some top-cited polyclonal antibodies may not be available through these partners.

Secondly, the YCharOS effort employs a non-biased approach that is agnostic to the protein for which antibodies have been characterized. The aim is to provide objective data on antibody performance without preconceived notions about how antibodies should perform or the molecular weight that should be observed in western blot. As the authors are not experts in STXBP1, only a brief overview of the protein’s function and its relevance in disease is provided. STXBP1 experts are invited to analyze and interpret the observed banding pattern in western blot and subcellular localization in immunofluorescence.

Thirdly, YCharOS experiments are not performed in replicates primarily due to the use of multiple antibodies targeting various epitopes. Once a specific antibody is identified, it validates the protein expression of the intended target in the selected cell line, confirms the lack of protein expression in the KO cell line and supports conclusions regarding the specificity of the other antibodies. All experiments are performed using master mixes, and meticulous attention is paid to sample preparation and experimental execution. In IF, the use of two different concentrations serves to evaluate antibody specificity and can aid in assessing assay reliability. In instances where antibodies yield no signal, a repeat experiment is conducted following titration. Additionally, our independent data is performed subsequently to the antibody manufacturers internal validation process, therefore making our characterization process a repeat.

Lastly, as comprehensive and standardized procedures are respected, any conclusions remain confined to the experimental conditions and cell line used for this study. The use of a single cell type for evaluating antibody performance poses as a limitation, as factors such as target protein abundance significantly impact results. ^6^ Additionally, the use of cancer cell lines containing gene mutations poses a potential challenge, as these mutations may be within the epitope coding sequence or other regions of the gene responsible for the intended target. Such alterations can impact the binding affinity of antibodies. This represents an inherent limitation of any approach that employs cancer cell lines.

## Method

The standardized protocols used to carry out this KO cell line-based antibody characterization platform was established and approved by a collaborative group of academics, industry researchers and antibody manufacturers. The detailed materials and step-by-step protocols used to characterize antibodies in western blot, immunoprecipitation and immunofluorescence are openly available on Protocol Exchange, a preprint server (DOI: 10.21203/rs.3.pex-2607/v1). ^6^ Brief descriptions of the experimental setup used to carry out this study can be found below.

### Cell lines and antibodies

Cell lines used and primary antibodies tested in this study are listed in Tables 1 and 2, respectively. To ensure that the cell lines and antibodies are cited properly and can be easily identified, we have included their corresponding Research Resource Identifiers, or RRID. ^10, 11^

**Table 2.** Summary of the STXBP1 antibodies tested.

Company	Catalog number	Lot number	RRID (Antibody Registry)	Clonality	Clone ID	Host	Concentration (μg/μL)	Vendors recommended applications
Abcam	ab109023 **	1041289-12	AB_10861492	recombinant mono	EPR4849	rabbit	1.90	Wb, IF
Abcam	ab124920 **	1002383-3	AB_10976239	recombinant mono	EPR4850	rabbit	0.11	Wb
Abcam	ab315893 **	1078682-6	AB_3097795	recombinant mono	EPR27956-86	rabbit	0.49	Wb
ABclonal	A5420	0014490101	AB_2766228	polyclonal	-	rabbit	1.01	Wb, IF
Bio-Techne (Novus Biologicals)	NBP3-15097 **	230461	AB_3094817	recombinant mono	S05-4B6	rabbit	n/a	Wb, IF
Bio-Techne (R&D Systems)	AF5675	CCWY0221041	AB_2302664	polyclonal	-	goat	0.20	Wb
Cell Signaling Technology	13414 **	1	AB_2798213	recombinant mono	D4O6V	rabbit	0.05	Wb
GeneTex	GTX114808	40233	AB_10620724	polyclonal	-	rabbit	0.80	Wb, IF
GeneTex	GTX114809	40233	AB_10620462	polyclonal	-	rabbit	0.96	Wb, IF
Proteintech	67137-1-Ig *	10009641	AB_2882436	monoclonal	1B5B3	mouse	2.50	Wb, IF
Thermo Fisher Scientific	MA5-35796 **	YE3913561B	AB_2849696	recombinant mono	ARC1518	rabbit	0.20	Wb
Thermo Fisher Scientific	PA1-742	WL342947	AB_325854	polyclonal	-	rabbit	1.00	Wb, IP, IF

Wb = western blot; IF = immunofluorescence; IP = immunoprecipitation; n/a = not available.

* Monoclonal antibody.

** Recombinant antibody.

**Table 3.** Illustrations to assess antibody performance in all western blot, immunoprecipitation and immunofluorescence.

Western blot	Immunoprecipitation	Immunofluorescence
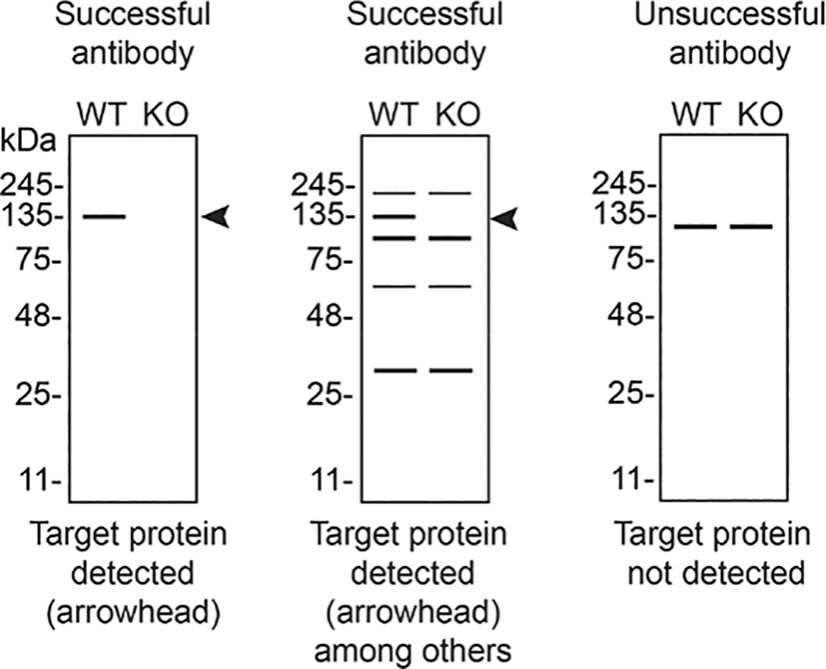	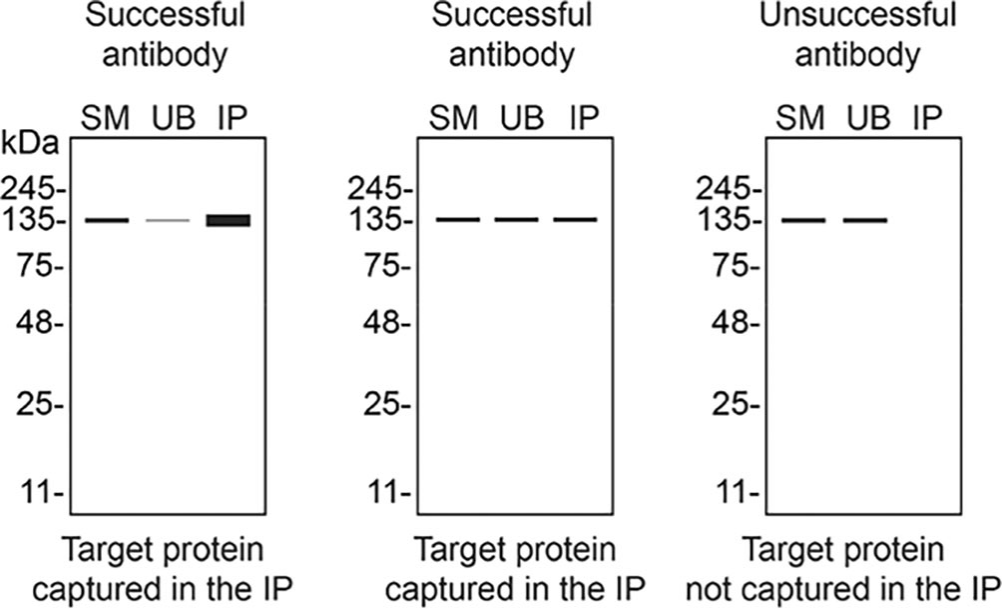	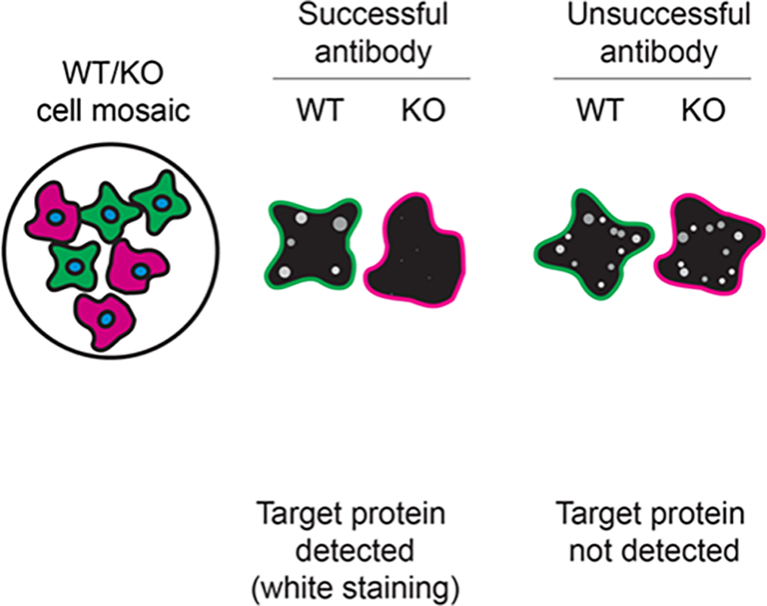

This table has been reproduced with permission from Ayoubi et al., Elife, 2023. ^3^

CRISPR/Cas9 genome editing: U-87 MG KO clone corresponding to the *STXBP1* gene was generated at Abcam. Two guide RNAs were used to induce a deletion in exon 3 of the *STXBP1* gene (sequence guide 1: TGGTGGATCAGTTAAGCATG, sequence guide 2: ATGACAGACATCATGACCGA).

Peroxidase-conjugated goat anti-rabbit and anti-mouse antibodies are (Thermo Fisher Scientific, cat. number 65-6120 and 62-6520). Peroxidase-conjugated donkey anti-goat antibody is from Thermo Fisher Scientific (cat. number A15999). Alexa-555-conjugated goat anti-rabbit and anti-mouse secondary antibodies (Thermo Fisher Scientific, cat. number A-21429 and A-21424). Alexa-555-conjugated donkey anti-goat secondary antibody is from Thermo Fisher Scientific (cat. number A-21432). Peroxidase-conjugated Protein A for IP detection is from Cell Signaling Technology, cat. number 12291.

### Antibody screening by western blot

U-87 MG WT and *STXBP1* KO cells were collected in RIPA buffer (25mM Tris-HCl pH 7.6, 150 mM NaCl, 1% NP-40, 1% sodium deoxycholate, 0.1% SDS) (Thermo Fisher Scientific, cat. number 89901) supplemented with 1x protease inhibitor cocktail mix (MilliporeSigma, cat. number P8340). Lysates were sonicated briefly and incubated 30 min on ice. Lysates were spun at ~110,000 × *g* for 15 min at 4°C and equal protein aliquots of the supernatants were analyzed by SDS-PAGE and western blot. BLUelf prestained protein ladder (GeneDireX, cat. number PM008-0500) was used.

Western blots were performed with precast midi 4-20% Tris-Glycine polyacrylamide gels (Thermo Fisher Scientific, cat. number WXP42012BOX) ran with Tris/Glycine/SDS buffer (Bio-Rad, cat. number 1610772), loaded in Laemmli loading sample buffer (Thermo Fisher Scientific, cat. number AAJ61337AD) and transferred on nitrocellulose membranes. Proteins on the blots were visualized with Ponceau S staining (Thermo Fisher Scientific, cat. number BP103-10) which is scanned to show together with individual western blot. Blots were blocked with 5% milk for 1 hr, and antibodies were incubated O/N at 4°C with 5% milk in TBS with 0.1% Tween 20 (TBST) (Cell Signalling Technology, cat. number 9997). Following three washes with TBST, the peroxidase conjugated secondary antibody was incubated at a dilution of ~0.2 μg/ml in TBST with 5% milk for 1 hr at room temperature followed by three washes with TBST. Membranes were incubated with Pierce ECL (Thermo Fisher Scientific, cat. number 32106) or Clarity Western ECL Substrate (Bio-Rad, cat. number 1705061) prior to detection with the iBright™ CL1500 Imaging System (Thermo Fisher Scientific, cat. number A44240).

### Antibody screening by immunoprecipitation

U-87 MG WT were collected in Pierce IP lysis buffer (Thermo Fisher Scientific, cat. number 87788) (25 mM Tris-HCl pH 7.4, 150 mM NaCl, 1 mM EDTA, 1% NP-40 and 5% glycerol) supplemented with protease inhibitor. Lysates were rocked 30 min at 4°C and spun at 110,000 × *g* for 15 min at 4°C.

Immunoprecipitation was performed using the KingFisher Apex purification system from Thermo Fisher Scientific (cat. number 5400930). Briefly, 2 μg of antibody were conjugated to 30 μl of Dynabeads protein A- (for rabbit antibodies) or protein G- (for mouse and goat antibodies) (Thermo Fisher Scientific, cat. number 10002D and 10004D, respectively) in 500 μl of Pierce IP Buffer. Conjugation was allowed for 30 min at 4°C followed by two 30 sec washes to remove unbound antibodies. Antibody NBP3-15097** is at an unknown concentration and therefore 5 μl were tested in the IP.

0.4 ml aliquots at 1.5 mg/ml of lysate were incubated with an antibody-bead conjugate for 1h at 4°C and beads were subsequently washed three times for 30 sec in 1.0 ml of IP buffer. All steps were scripted with recurring loops of mixing to keep the beads in suspension. Elution was set at 65°C for 10 min. Unbound fractions and eluates were collected at the end of the run and processed for SDS-PAGE and western blot on precast midi 4-20% Tris-Glycine polyacrylamide gels. Protein A:HRP was used as a secondary detection system at a concentration of 0.5 μg/ml.

### Antibody screening by immunofluorescence

U-87 MG WT and *STXBP1* KO cells were labelled with a green and a far-red fluorescence dye, respectively (Thermo Fisher Scientific, cat. number C2925 and C34565). The nuclei were labelled with DAPI (Thermo Fisher Scientific, cat. Number D3571) fluorescent stain. WT and KO cells were plated on 96-well plate with optically clear flat-bottom (Perkin Elmer, cat. number 6055300) as a mosaic and incubated for 24 hrs in a cell culture incubator at 37 ^o^C, 5% CO _2_. Cells were fixed in 4% paraformaldehyde (PFA) (VWR, cat. number 100503-917) in phosphate buffered saline (PBS) (Wisent, cat. number 311-010-CL). Cells were permeabilized in PBS with 0.1% Triton X-100 (Thermo Fisher Scientific, cat. number BP151-500) for 10 min at room temperature and blocked with PBS with 5% BSA, 5% goat serum (Gibco, cat. number 16210-064) and 0.01% Triton X-100 for 30 min at room temperature. Cells were incubated with IF buffer (PBS, 5% BSA, 0.01% Triton X-100) containing the primary STXBP1 antibodies overnight at 4°C. Cells were then washed 3 × 10 min with IF buffer and incubated with corresponding Alexa Fluor 555-conjugated secondary antibodies in IF buffer at a dilution of 1.0 μg/ml for 1 hr at room temperature with DAPI. Cells were washed 3 × 10 min with IF buffer and once with PBS.

Images were acquired on an ImageXpress micro confocal high-content microscopy system (Molecular Devices), using a 20x NA 0.95 water immersion objective and scientific CMOS cameras, equipped with 395, 475, 555 and 635 nm solid state LED lights (lumencor Aura III light engine) and bandpass filters to excite DAPI, Cellmask Green, Alexa-555 and Cellmask Red, respectively. Images had pixel sizes of 0.68 × 0.68 microns, and a z-interval of 4 microns. For analysis and visualization, shading correction (shade only) was carried out for all images. Then, maximum intensity projections were generated using 3 z-slices. Segmentation was carried out separately on maximum intensity projections of Cellmask channels using CellPose 1.0, and masks were used to generate outlines and for intensity quantification. ^12^ Figures were assembled with Adobe Illustrator.

## Data Availability

Zenodo: Antibody Characterization Report for STXBP1, https://doi.org/10.5281/zenodo.13891600. ^13^ Zenodo: Dataset for the STXBP1 antibody screening study, https://doi.org/10.5281/zenodo.14481229. ^14^ Data are available under the terms of the Creative Commons Attribution 4.0 International license (CC-BY 4.0).
