# Supplementary material for: A guide to selecting high-performing antibodies for STXBP1 (UniProt ID: P61764) for use in western blot, immunoprecipitation, and immunofluorescence
Source: F1000Res. 2024 Dec 24;13:1557. [Version 1] doi: 10.12688/f1000research.160174.1 (PMC12949838; doi:10.12688/f1000research.160174.1)
Supplement: Peer review history

# Reviewer report: Reviewer response for version 1

**DOI:** 10.5256/f1000research.176020.r458327

**Published:** 2026-02-28

**Authors:** Deborah Moshinsky

**Affiliations:** 1 Institute for Protein Innovation, Boston, Massachusetts, USA

The Syntaxin-binding protein 1, STXBP1, is involved in docking and fusion of synaptic vesicles, a crucial event for neurotransmitters release into the synapse.  The authors tested 12 commercially available antibodies to STXBP1 for activity in Western Blotting, IP, and IF using cells expressing the antigen vs. the same cell line with the antigen knocked out.  The standardized protocols are clearly described and the data are clearly presented.  For each test application, renewable antibodies were found that specifically recognize STXBP1.  This study should act as a useful guide for anyone who requires guidance in selecting an appropriate STXBP1 for use in Western Blot, IP or IF studies.

Are sufficient details of methods and materials provided to allow replication by others?

Yes

Is the rationale for creating the dataset(s) clearly described?

Yes

Are the datasets clearly presented in a useable and accessible format?

Yes

Are the protocols appropriate and is the work technically sound?

Yes

Reviewer Expertise:

Antibody Characterization and Validation

I confirm that I have read this submission and believe that I have an appropriate level of expertise to confirm that it is of an acceptable scientific standard.

# Reviewer report: Reviewer response for version 1

**DOI:** 10.5256/f1000research.176020.r358321

**Published:** 2025-01-16

**Authors:** Lynne Howells

**Affiliations:** 1 University of Leicester, Leicester, UK

This article describes the characterisation of recombinant/monoclonal Syntaxin-binding protein 1 (STXBP1) antibodies across 3 different platforms - namely western blot, immunoprecipitation and  immunofluorescence. The purpose is to allow investigators to choose high performing, pre-characterised antibodies to facilitate reproducible research into their protein of choice.

 This is a well written article using an established validation platform. There are a number of caveats to the data shown (ie, use of recombinant/monoclonals only, single replicates) but these are adequately described and justified. The delivery of the immunofluorescence platform is particularly well thought out.

 Minor comments: it would be useful to comment on the expected localisation of the protein in the IF platform (eg intracellular, nuclear, punctate etc). Please use capital 'L' in micro Litres rather than ml.

Are sufficient details of methods and materials provided to allow replication by others?

Yes

Is the rationale for creating the dataset(s) clearly described?

Yes

Are the datasets clearly presented in a useable and accessible format?

Yes

Are the protocols appropriate and is the work technically sound?

Yes

Reviewer Expertise:

Cancer biology

I confirm that I have read this submission and believe that I have an appropriate level of expertise to confirm that it is of an acceptable scientific standard.
